# Effects of the Different Dietary Fish Oil Calcium Salt on Production Performance, Blood Biochemical Parameters, Egg Quality Traits and Yolk Fatty Acid Profile in Laying Hens at Peak Production Period

**DOI:** 10.1002/vms3.70610

**Published:** 2025-10-27

**Authors:** Hamze Ghaderi‐Chaparabad, Seyyed Ali Mirghelenj, Sina Payvastegan, Hamed Khalilvandi‐Behroozyar, Amir Mosayyeb Zadeh

**Affiliations:** ^1^ Department of Animal Science, College of Agriculture and Natural Resources Urmia University Urmia Iran

**Keywords:** calcium salt, egg enrichment, fish oil, laying hen, omega‐3 fatty acids

## Abstract

**Background:**

Consuming foods containing appropriate levels of omega‐3, specifically eicosapentaenoic acid and docosahexaenoic acid, has been recommended for many years to prevent cardiovascular and nerve system disease or their related morbidity and mortality.

**Objectives:**

This study aimed to investigate the effects of different dietary fish oil calcium salt (FOCS) supplementation on production performance, blood biochemical parameters, egg quality traits and fatty acid (FA) profile of the egg yolk in laying hens at peak production period.

**Methods:**

In total, 192 45‐week‐old Hy‐Line W80 laying hens were randomly assigned into 4 treatments, 6 replicates and 8 birds each with similar initial body weight (1593 ± 65 g) for a consecutive 8 weeks. The experimental treatments, based on the corn–soybean meal, were as follows: (1) control (basal diet; CON); (2) 0.75% FOCS (FOCS_0.75_); (3) 1.5% FOCS (FOCS_1.5_) and (4) 2.25% FOCS (FOCS_2.25_).

**Results:**

The results of the current study indicated that egg production (EP), egg weight (EW) and egg mass (EM) increased significantly in FOCS‐treated birds compared to the CON group during the first 4 weeks and the whole experimental period. Blood not‐esterified fatty acids (NEFA), serum malondialdehyde (MDA) and uric acid (UA) decreased by FOCS supplementation (*p* < 0.05). The blood calcium of FOCS_2.25_ and the phosphorus of FOCS_1.5_ and FOCS_2.25_ groups were significantly reduced. At Week 8 of the experiment, shell thickness increased in the FOCS_2.25_ group (*p* < 0.05). FA profile analysis showed that polyunsaturated FAs (PUFAs), especially *n*‐3 PUFAs, increased and saturated FAs (SFAs) decreased by FOCS supplementation (*p* < 0.05).

**Conclusions:**

Dietary supplementation with FOCS_0.75_ is recommended to improve production performance and egg quality traits in laying hens during peak production. Moreover, FOCS_2.25_ can be used to enrich eggs with *n*‐3 PUFAs without adverse effects on bird health and egg quality.

## Graphical abstract

Dietary supplementation with fish oil calcium salt improved laying performance, yolk fatty acid profile and egg quality traits during the peak production period. Higher inclusion levels significantly increased *n*‐3 PUFA content without negative effects on bird health, while moderate levels (0.75%) optimized performance and productivity.

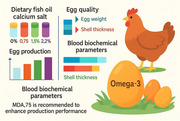

## 1 Introduction

The nutrient requirements of commercial egg‐producing hens, especially for energy, change as they age from the onset of laying onwards. This is mainly due to their high feed intake (FI) potential (Abou‐Kassem et al. 2019). Younger birds have shown high nutrient (energy) demands, compared to the elder ones (DePersio et al. 2015), in order to support either the egg production (EP) percentage or egg weight (EW) at the early production stage. In this regard, studies showed that laying hens respond well to increasing dietary energy levels or even increasing dietary fat inclusion (Sohail et al. 2003; Wu et al. 2007, 2005). In their most recent study, Kazemi et al. (2022) proved that almost 2%–5% dietary energy, and therefore nutrient concentration, above the nutrient recommendations of Hy‐Line W‐36 strain will result in improved production performance without negative effects on egg quality during the peak production period.

Although the production performance has recently been shown to be less affected by different dietary fat sources (Moran et al. 2019; Huang et al. 2020; Kralik et al. 2021), there are reports of increased EP and EW by different dietary fat sources (Cufadar et al. 2016; Aguillón‐Páez et al. 2020). Egg yolk FA profile and blood biochemical contents alterations have also varied according to dietary fat source (Ceylan et al. 2011; Irawan et al. 2022). For instance, in their most recent meta‐analysis study, Irawan et al. (2022), summarizing the findings of feeding laying hens with different dietary fat sources, indicated that yolk FA profile, specifically the *n*‐3 polyunsaturated FAs (PUFAs) contents, linearly correlates with the total *n*‐3 PUFAs, ALA and LA:ALA of dietary fat source with fish oil resulting in the highest *n*‐3 PUFAs compared to the others.

Consuming foods containing appropriate levels of *n*‐3 PUFAs, specifically eicosapentaenoic acid (EPA) and docosahexaenoic acid (DHA), has been recommended for many years to prevent cardiovascular and nerve system disease (Mason et al. 2020) or their related morbidity and mortality (Khan et al. 2021). Pointing out the antiarrhythmic, antithrombotic and anti‐inflammatory features of EPA and DHA, Fraeye et al. (2012) stated that these impacts may be attributed to the membrane structure stabilization and triglyceride (TG)‐reducing ability of these essential FAs in the human body. Hence, there has been a growing desire for foods containing *n*‐3 PUFAs (EPA and DHA), whereas the consumption of fish and fish oil as potential sources of EPA and DHA has decreased. This decline is due to the decrease in fish population caused by water pollution from heavy metals and polychlorinated biphenyls (Domingo and Bocio 2007). Therefore, consuming foods enriched with *n*‐3 fatty acids (FAs) has been suggested as a potential way to ensure an adequate supply of DHA and EPA over extended periods (Molendi‐Coste et al. 2011). Due to its widespread availability and acceptability, enriching eggs has been identified as the most effective strategy so far.

Egg *n*‐3 FA enrichment has been well proven in recent studies, where the authors declared that egg yolk EPA, DHA and total *n*‐3 PUFAs increased as dietary fish oil supplementation increased up to 1.5% (alone or in combination with soy oil or microalgae) (Kralik et al. 2020, 2021). In their meta‐analysis study, Irawan et al. (2022) found that among all the *n*‐3 FA sources they evaluated, including fish oil, linseed oil, flaxseed oil and sunflower oil, fish oil supplementation showed the highest DHA levels. This emphasizes the potential for enriching eggs with *n*‐3 FAs through dietary fish oil supplementation. However, peroxidation issues are commonly associated with fish oils due to their high PUFA content, which highlights the necessity of the preservatory practices. Soap formation in the gut (through a reaction with phosphoric acid, which results in the production of phospholipids that are often excreted along with biliary pigments) (Dumont and Narine 2007), low mixability (Rising et al. 1990), peroxidation during long‐term storage (Shahryari et al. 2021) and so forth are some of the profound problems associated with *n*‐3 PUFA, specifically fish oil, usage in poultry feeds.

Calcium salt (CS) of *n*‐3 PUFA‐rich fat sources has been shown to solve most of these problems. However, few, promising reports have been made by dietary CS supplementation of different fat sources. Promising results, including improved % EP, increased hatchability, economic profit, increased egg yolk DHA and EPA and reduced *n*‐6:*n*‐3 ratio, have been reported by feeding 1.5% either fish oil calcium salt (FOCS) or soy oil CS (SOCS) supplementation in aged broiler breeders (Sattari Najaf Abadi et al. 2021). Additionally, in 32‐week‐old laying hens, supplementation of wheat‐based diets with 3%, 6% and 9% animal fat CS along with 10% calcium carbonate led to an improvement in dietary true metabolizable energy (ME) (Rising et al. 1990). In another attempt, 1% black soldier fly larvae CS supplementation increased mono‐unsaturated FA (MUFA) and medium‐chain FAs while decreasing abdominal fat pad, hepatic FA synthase gene expression and total saturated FA (SFA) content of broiler chicken meat (Al Anas et al. 2024). Furthermore, Shahryari et al. (2021) also reported increased intestinal villus height and reduced villus height to crypt depth ratio, resulting in improved dry matter and protein digestibility, enhanced feed efficiency and increased body weight gain in broiler chickens fed with different levels of dietary SOSC.

On the basis of the limited reports available, this experiment was designed to investigate the effects of different dietary FOCS supplementations on production performance, egg quality traits and blood biochemical contents, while also evaluating egg yolk FA profile alterations in laying hens at their peak production period.

## 2 Materials and Methods

### 2.1 Chemicals

Chemical substances of Merck (Darmstadt, Germany) and Sigma Aldrich (St. Louis, MO) companies were used for all the laboratory determinations in the current work.

### 2.2 Birds, Housing and Fish Oil Calcium Salt (FOCS)

A total of 192 Hy‐Line W80 laying hens, 45 weeks old, with similar initial body weights (1593 ± 65 g) were divided into four treatments, with six replicates and eight birds each, in a completely randomized design. Ad libitum access to fresh water was provided, with an environmental temperature of 20–24°C, ambient moisture of 40%–50%, 16 h of light (max 30 lux intensity) and 8 h of dark (max 3 lux intensity) throughout the study. The FOCS was provided by Kimia Danesh Alvand Co. (the manufacturer of fat powders pure and calcium, Qom, Iran—PERSIA FAT) and supplemented to the corn–soybean meal‐based diets that were formulated by UFFDA software in accordance with the nutritional recommendations of Hy‐Line (2020). Experimental treatments were as follows: (1) control (CON, basal diet), (2) 0.75% FOCS (FOCS_0.75_), (3) 1.5% FOCS (FOCS_1.5_) and (4) 2.25% FOCS (FOCS_2.25_) supplemented diets that were fed to the birds twice daily at 8:00 AM and 2:00 PM, with each bird receiving 105 g/day (Table 1). The matrix value of the provided FOCS was determined earlier (Table 2) and taken into consideration during the feed formulation. In this study, the birds were initially fed a basal diet (without FOCS supplementation) for 2 weeks to allow for nutritional adaptation. Following this adaptation period, the experimental diets were administered for 8 weeks.

**TABLE 1:** Feed ingredients and nutrient composition of the experimental diets.

Ingredients (%)	CON	FOCS_0.75_	FOCS_1.5_	FOCS_2.25_
Corn	57.48	56.51	55.54	54.03
Soybean meal (44%)	29.85	30.07	30.29	30.63
FOCS	0	0.75	1.5	2.25
DCP	1.89	1.89	1.89	1.89
Calcium carbonate	9.57	9.36	9.21	9.10
Common salt	0.35	0.35	0.35	0.35
Sodium bicarbonate	0.1	0.1	0.1	0.1
DL‐methionine	0.16	0.17	0.17	0.17
Vitamin premix^a^	0.3	0.3	0.3	0.3
Mineral premix^b^	0.3	0.3	0.3	0.3
Calculated nutrient composition				
Metabolizable energy (kcal/kg)	2740	2760	2780	2800
Crude protein (%)	16.7	16.7	16.7	16.7
Calcium (%)	4.15	4.15	4.15	4.20
Available phosphorus (%)	0.48	0.48	0.48	0.48
Crude fibre (%)	3.44	3.43	3.43	3.42
Sodium (%)	0.18	0.18	0.18	0.18
Methionine (%)	0.45	0.45	0.45	0.45
Methionine + cysteine (%)	0.76	0.76	0.76	0.76
Lysine (%)	0.92	0.94	0.95	0.96
Threonine (%)	0.68	0.68	0.68	0.69
Tryptophan (%)	0.26	0.26	0.26	0.26
DCAB (mEq/kg)	206	206	207	207

Abbreviations: CON, control group; DCAB, dietary cation–anion balance; DCP, dicalcium phosphate; FOCS, fish oil calcium salt; FOCS_0.75_, 0.75% fish oil calcium salt supplemented group; FOCS_1.5_, 1.5% fish oil calcium salt supplemented group; FOCS_2.25_, 2.25% fish oil calcium salt supplemented group.

^a^ Supplied vitamins per kilogram of diet: A 10,000 IU, D_3_ 2500 IU, E 10 IU, B_1_ 2.2 mg, B_2_ 4 mg, B_3_ 8 mg, B_6_ 2 mg, B_9_ 0.56 mg, B_12_ 0.015 mg, choline 200 mg.

^b^ Supplied minerals per kilogram of diet: Mn 80 mg, Fe 50 mg, Zn 60 mg, Cu 12 mg, sodium selenite 0.3 mg.

**TABLE 2:** Fatty acid profile of the current fish oil calcium salt.

Indices	Unit	Test result	Test method
Total fat matter	In dry matter	78.85	AOAC
Ash	%	16.05	AOAC
Moisture	%	3.5	AOAC
Peroxide value	MeqO_2_/g	≤0.5	Titration
C14:0	% of total fatty acids	2.1	Gas chromatography
C16:0		24.5	
C16:1		4.2	
C18:0		4.85	
C18:1		34.2	
C18:2		10.21	
C18:3		1.46	
C20:5		5.8	
C22:6		12.2	

### 2.3 Production Performance

Daily hen‐day EP % and mean EW (g) were determined alongside the weekly egg mass (EM), FCR and FI (g). In this context, the % of EP was calculated by dividing the number of eggs produced by the hen‐day value. The hen‐day value was obtained by multiplying the number of live birds by the duration of the experiment. The FCR was achieved by dividing daily FI by daily EM. The EM was calculated by multiplying the % of EP by the mean EW daily.

### 2.4 Egg Quality Traits

The quality traits of the egg were evaluated in the 4th and 8th weeks of the study, where 18 eggs per treatment (three eggs per replicate) were selected and stored at room temperature for 24 h. Briefly, the eggs were weighed using a 0.01 g electronic scale (model Shanghai Puchun Measure Instrument, Jy602, China). Various amounts of NaCl were dissolved in distilled water to create a solution for evaluating egg density, ranging from 1.0600 to 1.1250 (Butcher and Miles 1991). The density of the egg was determined by the solution in which the egg sank. A hydraulic egg‐breaking machine (model egg force reader, Ogawa Seiki, Japan) was used to measure the strength (kg/cm^2^) required to break an egg. The yolk and albumen of broken eggs were placed on a flat surface for Haugh unit evaluation. The albumen height was recorded where the tip of the Haugh meter ruler touched the albumen about 10 mm around the yolk. The Haugh unit was calculated on the basis of albumen height and EW in accordance with the related formula: Haugh unit = albumen height − (1.7 × EW^0.37^) + 7.57 (Haugh 1937). The yolk and albumen were then weighed separately using the same scale mentioned earlier. The pH of the yolk and albumen was measured using an electronic pH meter (model AZ‐8688 Detachable Pen Type; AZ Instrument Corporation, China) as outlined by Mosayyeb Zadeh et al. (2023). The height and diameter of the egg yolk were measured using the previously mentioned Haugh meter and calliper (0.01 mm, model Baking Win, China), respectively, and the obtained values were used to calculate the yolk index by dividing yolk height by its diameter. Yolk colour was determined optically using the Rosh fan colour scale. The eggshell was cleared and dried at room temperature for 72 h. Then, the whole shell was weighed, and the thickness of its three parts (top, middle and bottom) was measured.

### 2.5 Serum Biochemicals Parameters

Three blood samples per replicate (18 per treatment and at least 3.5 mL for each sample) were collected from the wing vein using a 5 mL plastic syringe and placed at 45° angle at room temperature for 6 h to obtain serum samples. These serum samples were then centrifuged at 5600 *g* for 12 min at 18°C in a 1.5 mL microtube to obtain a better and clearer sample, before being transferred to the lab at −20°C. In the laboratory (Department of Drug Applied Research Center, Tabriz University of Medical Sciences, Tabriz, Iran), the serum biochemicals were quantitatively determined using the kits provided by Pars Azmoon and an autoanalyser (model Technicon RA 1000); Bayer The methods and materials used to determine total antioxidant capacity (TAC), malondialdehyde (MDA), ALT, aspartate aminotransferase (ASP), alkaline phosphatase (ALP), albumin and lipids such as cholesterol and TGs are described in the study by Mosayyeb Zadeh et al. (2023). Serum not‐esterified fatty acid (NEFA) content was measured using the kits provided by RX MONZA following the manufacturer's instructions. In this process, 50 µL of serum sample (along with standard, blank and sample blank) was mixed with 1 mL of reagent 1 solution at 37°C for 10 min. The obtained solution was mixed with 2 mL of reagent 2 and incubated at 37°C followed by recording light absorbance at 550 nm of wavelength against a blank in autoanalyser. The NEFA content was then determined using the formula outlined in the kit's procedure guideline. The serum glucose was measured using kits provided by Pars Azmoon, based on the glucose oxidase‐catalysed (GOD) reaction. For this, 10 µL of serum and standard solutions were mixed with 1 mL of the reagent (consisting of phosphate buffer [pH 7.5], phenol, 4‐aminoantipyrine, glucose oxidase, and peroxidase) and incubated for 10 min at 37°C. The light absorbance at 546 nm wavelength was then read and compared with a blank (distilled water) solution. The uric acid (UA) was determined according to the common TOOS (*N*‐ethyl‐*N*‐(hydroxy‐3‐sulphopropyl)‐m‐toluidin) reaction method. The reagents 1 and 2 were prepared as described by manufacture instructions, and the light absorbance of sample and standard at 546 nm of wavelength was compared with that of blank solution. The UA was finally calculated using the formula described in provided kit instructions (Pars Azmoon). The serum calcium level was determined using the o‐Cresolphthalein Complexone method. Initially, 20 µL of serum sample was mixed with 1 mL of reagent one (ethanolamine detergent pH 10.7), and after 5 to maximum 30 min, the light absorbance was measured alongside a standard solution (reagent one + distilled water). Subsequently, the sample and standard solutions were combined with 250 µL of reagent two, and the light absorbance of the resulting red‐coloured solution, which forms from the combination of calcium and cersolphthanine, was measured at a wavelength of 570 nm. The calcium content was then calculated using the formula provided by the manufacturer of the kits. For phosphorus determination (using the photometry/UV test method), 10 µL of serum sample was mixed with 1 mL of reagents 1 and 2 according to the Pars Azmoon kit instructions and then incubated for 5 min at 37°C. After the incubation, the light absorbance of the sample and standard solutions was measured at a wavelength of 360 nm and compared to that of the blank solution within 1 h.

### 2.6 FA Profile

The matrix value (FA profile) of the FOCS used in the present experiment was provided by the manufacturer (Kimia Danesh Alvand Co. [the manufacturer of fat powders pure and calcium, Qom, Iran]—PERSIA FAT), and the determination methods of each index have been illustrated in the mentioned table (Table 2). The lipids of experimental diets were extracted according to the method of chloroform:methanol mixture (2:1 v/v) as described by Floch (1957). In the case of yolk FA profile, the yolk samples of 18 eggs per treatment (three eggs per replicate) were collected at Week 8 of the experiment and stored at −20°C for FA profile determination at the University of Applied Science and Technology, Urmia, Iran. The yolk samples were extracted using the Soxhlet extraction method, homogenized by vortexing, and 100 mg of each sample was weighed. They were then saponified by adding 3 mL of 2 molar potassium hydroxide and esterified by adding 5 mL of 12% methanolic sulphuric acid. The methyl ester of FAs was injected into the gas chromatography machine (Agilent 6890, Agilent CO, USA) using 0.1 mL of extracted normal heptane and 1 µL of normal heptane phase. The gas chromatography system was equipped with a capillary injector, a specialized capillary column for FAs (Stable Wax, 30 m length, 0.32 mm internal diameter and 0.25 µm film thickness) and a flame ionization detector (FID). The initial oven temperature was set at 75°C for 1 min and gradually increased to 240°C (25°C per min) and maintained for 8 min (Yoon et al. 2024). Nitrogen gas (99.999% purity, provided by Oxygen Sabalan Co. [the UK 108 Air Product Company's agent]) was used as a carrier at a flow rate of 45 mL/min. The injector temperature was set at 250°C and the detector at 280°C. A mixture of all FAs’ standards was also injected, and its retention time was used for comparison with egg yolk FAs (Mannion et al. 2016). The results of the retention time comparison were analysed using ChemStation software in the Windows environment (Table 3).

**TABLE 3:** Fatty acid composition of experimental diets.

Fatty acid (% of total fatty acids)	CON	FOCS_0.75_	FOCS_1.5_	FOCS_2.25_
C14:0	0.09	0.011	0.012	0.012
C14:1	—	—	—	—
C16:0	27.09	31.97	34.82	37.45
C16:1	0.07	0.056	0.045	0.047
C18:0	3.91	4.11	4.18	5.09
C18:1 *n*‐9	31.66	26.88	25.39	23.33
C18:1 *n*‐7	0.67	0.55	0.53	0.50
C18:2 *n*‐6	35.01	33.30	31.25	29.18
C18:3 *n*‐3	0.31	0.31	0.39	0.39
C20:4 *n*‐6	0.33	0.35	0.36	0.42
C20:5 *n*‐3	0.01	0.63	0.79	0.88
C22:6 *n*‐3	0.01	0.71	0.81	0.92
SFA	31.01	36.02	39.11	42.54
PUFA	35.66	35.01	33.46	31.69
PUFA:SFA	1.15	0.972	0.858	0.745
*n*‐3	0.33	1.35	1.85	2.09
*n*‐6	35.33	33.65	31.61	29.60
*n*‐6:*n*‐3	107.1	24.92	17.08	14.16
LA:ALA	112.9	107.4	80.12	74.82

Abbreviations: CON, control group; FOCS_0.75_, 0.75% fish oil calcium salt supplemented group; FOCS_1.5_, 1.5% fish oil calcium salt supplemented group; FOCS_2.25_, 2.25% fish oil calcium salt supplemented group.

### 2.7 Statistical Analysis

The data from the current experiment were analysed using the GLM procedure in SAS software version 9.2 (SAS Support 2009). To normalize percentage data (e.g., FA profile), the ArcSin√x transformation was applied. The statistical model used in this study was *y_ij_* = *μ* + *T_i_* + *e_ij_*, where *y_ij_* represented the observations, *μ* was the mean of the observations, *T_i_* was the effect of FOCS, and *e_ij_* was the error of the observations. The significance of differences was evaluated using the Tukey test, and *p* < 0.05 was considered statistically different.

## 3 Results

### 3.1 Production Performance

The effects of dietary FOCS supplementation on the production performance of laying hens are shown in Table 4. As indicated, during the first 4 weeks, the EP increased in FOCS_0.75_ compared to the CON group, whereas during the whole experimental period, it increased linearly (*p* < 0.05). Except for the FOCS_2.25_ group at the first 4 weeks, the EW was increased linearly and quadratically by different dietary FOCS supplementations at the first 4 weeks and whole experimental periods compared to the CON group (*p* < 0.01). The FOCS_0.75_ and FOCS_1.5_ groups showed the highest EM during the first 4 weeks and the whole experimental period, where the changes were linearly and quadratically significant (*p* < 0.05). However, compared to the CON, the FCR decreased linearly and quadratically in the FOCS_0.75_ and FOCS_1.5_ groups during the first 4 weeks and whole experimental periods (*p* < 0.05).

**TABLE 4:** Effects of different dietary fish oil calcium salt (FOCS) supplementation on production performance of laying hens during peak production period.^*^

Indices	CON	FOCS_0.75_	FOCS_1.5_	FOCS_2.25_	SEM	*p* value	Polynomial comparisons	
							linear	quadratic
	First 4 weeks (1–4 weeks)							
EP (%)	90.80^b^	93.57^a^	92.94^ab^	91.87^ab^	0.594	0.022	0.21	0.33
EW (g)	52.95^b^	54.48^a^	54.45^a^	54.18^ab^	0.318	<0.01	<0.01	<0.01
FI (g)	103.0	102.4	102.8	103.6	0.374	0.19	0.71	0.291
EM (g)	48.08^b^	51.32^a^	50.63^a^	49.78^ab^	0.567	<0.01	<0.01	0.012
FCR (g:g)	2.14^a^	1.99^b^	2.03^b^	2.08^ab^	0.022	<0.01	<0.01	<0.01
	Second 4 weeks (4–8 weeks)							
EP (%)	91.25	91.96	92.41	92.14	0.528	0.47	0.139	0.839
EW (g)	55.55	56.32	56.61	56.62	0.352	0.946	0.051	0.581
FI (g)	102.8	103.2	103.0	103.0	0.469	0.946	0.766	0.608
EM (g)	50.7	51.8	52.31	52.18	0.618	0.277	0.083	0.698
FCR (g:g)	2.02	1.99	1.97	1.98	0.025	0.374	0.113	0.898
	Whole experimental period							
EP (%)	91.02^b^	92.77^a^	92.67^ab^	92.01^ab^	0.414	0.032	0.012	0.089
EW (g)	54.25^b^	55.58^a^	55.53^a^	55.40^a^	0.198	<0.01	<0.01	0.011
FI (g)	102.9	102.8	102.9	103.3	0.176	0.234	0.998	0.65
EM (g)	49.39^b^	51.56^a^	51.47^a^	50.98^ab^	0.399	<0.01	<0.01	0.034
FCR (g:g)	2.08^a^	1.99^b^	2.00^b^	2.02^ab^	0.016	<0.01	<0.01	0.027

*Note*: Means without a common superscript (a–c) differed (*p* < 0.05).

Abbreviations: CON, control group; EM, egg mass; EP, egg production; EW, egg weight; FCR, feed conversion ratio; FI, feed intake; FOCS_0.75_, 0.75% fish oil calcium salt supplemented group; FOCS_1.5_, 1.5% fish oil calcium salt supplemented group; FOCS_2.25_, 2.25% fish oil calcium salt supplemented group.

^*^ Values represent the means of 18 samples per treatment, and the samples were obtained at the end of the experiment.

### 3.2 Blood Biochemical Parameters

As indicated in Table 5, the blood NEFA contents decreased linearly and quadratically in the birds of FOCS_1.5_ and FOCS_2.25_ supplemented groups compared to those in FOCS_0.75_ and CON groups (*p* < 0.01). The MDA was highest and lowest in the FOCS_0.75_ and FOCS_2.25_ groups, respectively, compared to the CON, where the changes were shown to be quadratically significant (*p* < 0.01). The ALT increased linearly in all the FOCS‐supplemented groups (*p* < 0.01). The UA reduced linearly and quadratically in FOCS_0.75_ compared to the FOCS_1.5_ and FOCS_2.25_ groups (*p* < 0.05). Glucose increased linearly and quadratically in FOCS_1.5_ and FOCS_2.25_ compared to the FOCS_0.75_ and CON groups (*p* < 0.01). The blood calcium level decreased in the FOCS_2.25_ group, whereas its phosphorus reduced linearly and quadratically in the FOCS_1.5_ and FOCS_2.25_ groups (*p* < 0.01).

**TABLE 5:** Effects of different dietary fish oil calcium salt (FOCS) supplementation on blood biochemical of laying hens during peak production period.^*^

Indices	CON	FOCS_0.75_	FOCS_1.5_	FOCS_2.25_	SEM	*p* value	Polynomial comparisons	
							Linear	Quadratic
Cholesterol (mg/dL)	319.60	301.90	303.80	341.90	15.972	0.275	0.488	0.619
Triglyceride (mg/dL)	2546.0	2406.0	2417.9	2199.1	81.034	0.125	0.735	0.760
LDL (mg/dL)	491.20	481.20	483.40	439.80	16.206	0.125	0.735	0.760
HDL (mg/dL)	11.93	12.50	11.94	12.50	0.524	0.765	0.998	0.386
NEFA (mmol/L)	1.75^a^	1.62^a^	1.12^b^	1.17^b^	0.051	<0.01	<0.01	<0.01
TAC (mmol/L)	1.69	1.62	1.67	1.67	0.035	0.442	0.647	0.121
MDA (nmol/mL)	3.04^b^	3.54^a^	2.96^b^	2.45^c^	0.126	0.01	0.637	<0.01
ALT (IU/L)	35.90^b^	40.10^a^	40.10^a^	40.80^a^	0.893	<0.01	<0.01	0.062
AST (IU/L)	215.10	227.40	226.20	229.60	6.370	0.264	0.903	0.140
ALP (IU/L)	376.50	389.80	366.30	369.30	7.899	0.121	0.230	0.012
UA (mg/dL)	5.11^ab^	4.41^b^	6.33^a^	6.28^a^	0.376	<0.01	0.027	<0.01
Glu (mg/dL)	163.00^b^	172.00^b^	298.80^a^	293.60^a^	8.657	<0.01	<0.01	<0.01
Ca (mg/dL)	36.40^a^	36.80^a^	36.70^a^	30.70^b^	0.723	<0.01	0.770	0.779
P (mg/dL)	9.82^a^	10.47^a^	7.47^b^	8.02^b^	0.447	<0.01	<0.01	<0.01

*Note*: Means without a common superscript (a–c) differed (*p* < 0.05).

Abbreviations: ALP, alkaline phosphatase; ALT, alanine aminotransferase; AST, aspartate aminotransferase; Ca, calcium; CON, control group; FOCS_0.75_, 0.75% fish oil calcium salt supplemented group; FOCS_1.5_, 1.5% fish oil calcium salt supplemented group; FOCS_2.25_, 2.25% fish oil calcium salt supplemented group, LDL, low‐density lipoprotein; Glu, glucose; HDL, high‐density lipoprotein; MDA, malondialdehyde; NEFA, non‐esterified fatty acids; P, phosphorus; SEM, standard error of means; TAC, total antioxidant capacity; UA, uric acid.

^*^ Values represent the means of 18 samples per treatment, and the samples were obtained at the end of the experiment.

### 3.3 Egg Quality Traits

The results of feeding laying hens with different levels of FOCS‐supplemented diets on egg quality traits (Table 6) showed that at Week 4 of the experiment, the % eggshell increased linearly and quadratically (*p* < 0.05), the albumen pH decreased linearly in FOCS_2.25_ compared to the other experimental groups (*p* < 0.05), and the yolk pH was also reduced linearly in the FOCS_1.5_ and FOCS_2.25_ groups compared to the FOCS_0.75_ and CON (*p* < 0.05). At the eighth week of the experiment, egg Haugh unit was significantly reduced in the FOCS_2.25_ group compared to the CON group (*p* < 0.05). The yolk colour index increased linearly in FOCS_1.5_ and FOCS_2.25_ compared to the FOCS_0.75_ and CON groups (*p* < 0.01). However, at Week 8 of the experiment, eggshell thickness increased significantly in FOCS_2.25_ compared to the CON group (*p* < 0.05).

**TABLE 6:** Effects of different dietary fish oil calcium salt (FOCS) supplementation on egg quality traits of laying hens during peak production period.^*^

Indices	CON	FOCS_0.75_	FOCS_1.5_	FOCS_2.25_	SEM	*p* value	Polynomial comparisons	
							Linear	Quadratic
	Week 4 of the experiment							
Albumen (%)	60.35	60.38	59.40	59.39	0.612	0.301	0.101	0.508
Yolk (%)	27.39	26.47	27.69	27.25	0.557	0.116	0.107	0.403
Shell (%)	12.24^b^	13.15^a^	12.89^a^	13.36^a^	0.369	<0.01	<0.01	0.022
Albumen pH	6.54^a^	6.32^a^	5.84^a^	5.09^b^	0.189	<0.01	0.012	0.584
Yolk pH	6.40^ab^	6.49^a^	5.78^b^	4.92^c^	0.182	<0.01	0.021	0.080
Haugh unit	84.93	88.26	87.18	87.55	2.429	0.138	0.097	0.165
Shell thickness (mm)	0.459	0.461	0.473	0.479	0.011	0.605	0.904	0.369
Shell strength (N/m^2^)	3.96	3.69	4.54	4.55	0.316	0.156	0.203	0.157
Yolk colour	5.90^b^	6.00^b^	7.00^a^	7.80^a^	0.226	<0.01	<0.01	0.113
	Week 8 of the experiment							
Albumen (%)	58.95	58.82	58.82	58.08	0.462	0.441	0.093	0.324
Yolk (%)	27.46	28.31	27.84	28.35	0.542	0.229	0.258	0.181
Shell (%)	13.33	12.99	13.32	13.56	0.546	0.816	0.752	0.615
Albumen pH	8.49^b^	8.68^ab^	8.84^a^	8.61^ab^	0.095	0.086	0.013	0.932
Yolk pH	6.78	6.79	6.83	6.92	0.095	0.752	0.329	0.828
Haugh unit	91.81^a^	91.49^ab^	91.15^ab^	90.91^b^	0.439	0.043	0.037	0.547
Shell thickness (mm)	0.438^b^	0.473^ab^	0.467^ab^	0.483^a^	0.011	0.039	0.067	0.173
Shell strength (kg/cm^2^)	4.86	5.06	5.00	5.22	0.254	0.793	0.699	0.678
Yolk colour	5.60	5.60	5.50	5.30	0.161	0.520	0.664	0.802

*Note*: Means without a common superscript (a–c) differed (*p* < 0.05).

Abbreviations: CON, control group; FOCS_0.75_, 0.75% fish oil calcium salt supplemented group; FOCS_1.5_, 1.5% fish oil calcium salt supplemented group; FOCS_2.25_, 2.25% fish oil calcium salt supplemented group.

^*^ Values represent the means of 18 samples per treatment.

### 3.4 Yolk FA Profile

The effects of dietary treatments on the yolk FA profile are indicated in Table 7. According to the results, C14:0, C18:1 (*n*‐7), and PUFA increased linearly (and quadratically in the case of PUFA) in the egg yolk of all FOCS‐treated birds compared to those of CON (*p* < 0.01). The yolk C18:2 (*n*‐6) content increased quadratically in FOCS_1.5_ and FOCS_2.25_ compared to the FOCS_0.75_ (*p* < 0.01). The C18:3 (*n*‐3) decreased linearly in the FOCS_1.5_ and FOCS_2.25_ groups compared to CON (*p* < 0.05). The C16:1, C20:4 (*n*‐6), C20:5 (*n*‐6), C22:6 (*n*‐3), *n*‐3 and PUFA:SFA ratio of egg yolk increased linearly and quadratically by increasing dietary levels of FOCS supplementation (*p* < 0.05). However, the *n*‐6:*n*‐3, LA:ALA, C18:0 and SFA contents decreased linearly (and quadratically in the case of *n*‐6:*n*‐3 ratio) by increasing dietary FOCS level in comparison to the CON group (*p* < 0.05). Moreover, C18:1 (*n*‐9) was reduced in egg yolk of all the FOCS‐supplemented groups compared to the CON (*p* < 0.01).

**TABLE 7:** Effects of different dietary fish oil calcium salt (FOCS) supplementation on yolk fatty acid profile of laying hens during peak production period.^*^

Indices	CON	FOCS_0.75_	FOCS_1.5_	FOCS_2.25_	SEM	*p* value	Polynomial comparisons	
							Linear	Quadratic
C14:0	0.328^b^	0.412^a^	0.424^a^	0.404^a^	0.017	<0.01	<0.01	0.118
C14:1	0.062	0.068	0.058	0.062	0.004	0.516	0.550	0.178
C16:0	25.39	25.34	25.22	24.62	0.312	0.308	0.702	0.930
C16:1	2.254^c^	2.998^b^	3.216^ab^	3.574^a^	0.089	<0.01	<0.01	0.029
C18:0	12.402^a^	11.130^b^	9.836^c^	9.560^c^	0.216	<0.01	<0.01	0.967
C18:1 *n*‐9	46.95^a^	44.80^b^	45.14^b^	45.28^b^	0.249	<0.01	<0.01	<0.01
C18:1 *n*‐7	0.240^b^	0.478^a^	0.492^a^	0.610^a^	0.048	<0.01	<0.01	0.078
C18:2 *n*‐6	10.29^ab^	10.83^a^	10.09^b^	9.73^b^	0.174	<0.01	0.438	<0.01
C18:3 *n*‐3	0.906^b^	0.964^ab^	1.000^a^	0.994^a^	0.020	0.049	0.013	0.712
C20:4 *n*‐6	0.162^d^	0.608^c^	0.884^b^	1.024^a^	0.021	<0.01	<0.01	<0.01
C20:5 *n*‐3	0.154^d^	0.558^c^	0.746^b^	1.042^a^	0.026	<0.01	<0.01	<0.01
C22:6 *n*‐3	0.308^d^	0.860^c^	1.234^b^	1.838^a^	0.025	<0.01	<0.01	0.013
SFA	38.12^a^	36.36^b^	35.48^bc^	34.58^c^	0.372	<0.01	<0.01	0.350
PUFA	11.82^b^	13.83^a^	13.96^a^	14.63^a^	0.199	<0.01	<0.01	0.014
PUFA:SFA	0.310^c^	0.378^b^	0.396^ab^	0.424^a^	0.008	<0.01	<0.01	0.023
*n*‐3	1.368^d^	2.382^c^	2.980^b^	3.874^a^	0.054	<0.01	<0.01	<0.01
*n*‐6	10.45^b^	11.44^a^	10.98^ab^	10.75^ab^	0.180	0.019	0.055	<0.01
*n*‐6:*n*‐3	7.662^a^	4.824^b^	3.690^c^	2.776^d^	0.134	0.014	<0.01	<0.01
LA:ALA	11.43^a^	11.27^ab^	10.11^bc^	9.79^c^	0.317	<0.01	<0.01	0.217

*Note*: Means without a common superscript (a–c) differed (*p* < 0.05).

Abbreviations: CON, control group; FOCS_0.75_, 0.75% fish oil calcium salt supplemented group; FOCS_1.5_, 1.5% fish oil calcium salt supplemented group; FOCS_2.25_, 2.25% fish oil calcium salt supplemented group; LA:ALA, linoleic acid to alpha–linoleic acid ratio; *n*‐3, omega‐3 fatty acids; *n*‐6, omega‐6 fatty acids; PUFA, polyunsaturated fatty acids; SFA, saturated fatty acids.

^*^ Values represent the means of 18 samples per treatment that were obtained at Week 10 of the experiment.

## 4 Discussion

As discussed earlier, due to its high acceptability, accessibility and potential for long‐term storage, enriching eggs with *n*‐3 PUFA has been considered the most effective strategy for producing foods containing essential FAs, particularly DHA and EPA (Molendi‐Coste et al. 2011). This allows consumers to benefit from their health‐promoting properties. This study was, therefore, an attempt to use dietary FOCS supplementation as a potential source of *n*‐3 PUFA to enrich eggs with DHA and EPA while evaluating its impact on production performance, egg quality and blood parameters of laying hens during the peak production period.

The results of the current study show that adding 0.75% and 1.5% FOCS to the diet improved the production performance of 45‐week‐old laying hens (Table 4). The EP, EW and EM of the hens receiving FOCS were significantly higher, and their FCR was lower compared to the control group during the first 4 weeks and throughout the whole study. As EM depends on EP and EW, increasing these factors is expected to result in higher EM. Additionally, the FCR is directly and indirectly influenced by FI and EM, respectively. Therefore, with constant FI, a lower FCR would be expected when EM increases. These findings are in agreement with the study of Sattari Najaf Abadi et al. (2021), who reported that dietary supplementation of 1.5% and 3% CS from the source of either fish oil or linseed, respectively, increased % EP and EW compared to the CON group. Those authors believed that the deviation in the biochemical pathways between fat synthesis and storage due to the energy oversupply resulted in energy efficacy pathways disturbance (through the activation of the glycolysis pathway by increasing the glucose‐6‐phosphatase activity (Turner et al. 1999)) which in turn reduced EP in higher CS supplementations. Studies have shown that a certain amount of linoleic acid (LA; C18:2 *n*‐6) is required to produce an egg. As the high dietary inclusion of *n*‐3 FA sources such as fish oil, linseed oil and sunflower oil reduces the LA:ALA ratio, the long‐term supplementation of fish oil or any *n*‐3 FA source with lower LA:ALA ratio will, therefore, decrease production performance, especially EW (Grobas et al. 1999). This study confirmed the assumption of previous researchers and indicated that increasing dietary FOCS supplementation significantly decreased LA and LA:ALA ratio. Therefore, in‐line with the previous investigations, the present authors believe that increasing the energy supply and providing the least LA required for EP may be responsible for the increased % EP and EW of the FOCS_0.75_ and FOCS_1.5_ compared to the CON at the first 4 weeks and whole experimental period. On the other hand, the lower % EP and EW in the FOCS_2.25_ group might be attributed to the energy oversupply and severe reduction of LA and LA:ALA ratio. However, Kralik et al. (2021) reported no significant effects of dietary 3.5% soy oil or 1.5% fish oil supplementation on laying hens’ production performance.

Corresponding to the current findings, feeding broiler chickens with soy oil acid or its CS resulted in non‐significant improvements in weight gain and FCR, with no changes in FI (Shahryari et al. 2021). The authors of the study confirmed earlier findings that increasing ME can improve performance and attributed the increased weight gain in broiler chickens fed soy oil acid or its CS to the effects of the fat source on intestinal development and increased feed retention time, leading to improved feed ingredient digestion and absorption. Additionally, they reported that broiler chickens fed SOCS showed improved feed efficiency compared to those fed soy oil acid, as they believed that soy oil acid increased abdominal fat deposition. This is significant because energy and nutrients used for fat deposition in the abdominal cavity are four times more than that of other tissues (Kleyn 2013), which could lead to reduced feed efficiency when broilers are fed soy oil acid‐supplemented diets. According to Shahryari et al. (2021), dietary SOCS supplementation increased villus height:crypt depth and decreased crypt depth at the end of the small intestine. The gastrointestinal tract plays a crucial role in digesting, absorbing and preventing pathogen permeation, utilizing almost 25% of the consumed energy (Hamard et al. 2010). Additionally, including dietary fat, especially CS, can enhance gut function and development by providing sufficient energy for enterocytes through slowing feed passage and continuing ingredient digestion and absorption until the end of the ileum (Swiatkiewicz et al. 2015). Therefore, dietary fat, especially CS, can improve bird performance by enhancing digestion, absorption and the health and development status of the intestine (Uni 2006; Samli et al. 2007; Shahryari et al. 2021). As mentioned earlier, CS reduces ME of the fat source; thus, in‐line with previous researchers, the current author believes that FOCS at a dietary level of 1.5% improved the production performance of laying hens, probably by providing appropriate energy and LA, along with improving intestinal morphology, digestion and absorption.

Shahryari et al. (2021) agreed with the previous studies (Corduk et al. 2007) that in broiler chickens selected for fast growth rate, improved performance parameters, especially meat yield and FCR, are likely due to the increased potential of using feed protein through the increase in dietary energy from different fat sources (Classen 2017). The findings of this study are consistent with previous research. Therefore, on the basis of the consistent FI during the first and second 4 weeks of the experiment and the improved performance parameters during the first 4 weeks when using FOCS_0.75_ and FOCS_1.5_ treatments, the authors believe that supplementing laying hens’ diet with either 0.75% or 1.5% FOCS may provide the necessary energy to maximize the use of feed protein during Weeks 47–51, the period when there is a high demand for energy and protein to support production performance. During the second 4 weeks of this study, the ages of the hens were between 52 and 56, which is considered the post‐peak production period. During this time, the % EP naturally declines. Although the % EP was higher than the CON group in the second 4 weeks, the differences were not statistically significant. The reason for this is not clear. However, it is interesting to note that during the same period, the EP of the control group increased, whereas the EP of all the FOCS‐supplemented groups decreased. This shows the consistency of the control group during the peak and post‐peak production periods. According to Grobas et al. (1999), increasing the percentage of EP (energy and protein) during the pre‐peak period by adding 4% dietary fat, increasing AMEn and maintaining dietary LA at 1.15% led to increased post‐peak period EP up to 65 weeks of age in Isa Brown laying hens. However, farm managers’ experiences with layers, including laying hens and broiler breeders, have shown that increasing the percentage of EP at peak production has resulted in decreased post‐peak production performance due to energy and calcium reserves depletion. The authors suggest that FOCS supplementation might be useful during the pre‐peak production period to support birds’ energy and calcium requirements. They encourage further investigations to study the effects of different dietary FOCS supplementation (at the same level as the present work) on performance, intestinal morphology and egg quality traits from the pre‐ to post‐peak production period.

In the current study, blood lipids were not affected by dietary treatments, except for the NEFA. Consistent with these findings, NEFA levels were shown to decrease when dietary sunflower oil was replaced with fish oil at the 1.5% and 3% dietary levels in 40‐week‐old New Hampshire laying hens (Hall et al. 2007). Besides, in‐line with the current results, no differences in blood cholesterol, TG and LDL were observed in 34‐week‐old laying hens fed 1.5% fish oil‐supplemented diets (Basmacioglu et al. 2003). The study mentioned above did not include the determination of blood NEFA levels. It is worth noting that almost 90% of the FAs needed by poultry are produced in the liver (Zhang et al. 2024). FAs are synthesized from precursors such as glucose and amino acid metabolites, and they ultimately contribute to the formation of TG (Kim et al. 2020). This process primarily takes place in hepatocytes with the involvement of acyl‐coenzyme A (CoA) and FA synthesis enzymes, including acyl‐CoA carboxylase (AAC) and fatty acid synthase (FAS) (Gu et al. 2022). Conversely, lipid transfer in the body involves the breakdown of TG structure by lipase and the generation of NEFAs (Wang et al. 2022). TG and NEFA are recognized as the main FA precursors, with nearly 40% of NEFAs being utilized for FA formation (Kadegowda et al. 2008; Ingraham et al. 2014). The liver is also known as the main tissue for TG and NEFA removal (Bell 1979). The liver plays a key role in processing nearly 80% of blood NEFAs. These NEFAs are either converted into energy in the form of ATP or stored in the liver as TG (Hocquette and Bauchart 1999; van Dorland et al. 2012). After entering the liver, NEFAs are either metabolized or distributed and then transferred to the tissues through the hepatic vein to be used in fat synthesis based on the tissues’ requirements. Previous research has demonstrated that PUFAs, particularly DHA and EPA found in fish oil, reduce hepatic fat synthesis (Wilson et al. 1986, 1990; Blake and Clarke 1990; Ntambi 1992; Sanz et al. 2000) and increase FA oxidation (Madsen et al. 1999; Sanz et al. 2000). Fish oil has been shown to reduce TG synthesizing enzymes, such as acyl‐CoA:1,2‐diacylglycerol *O*‐acyltransferase by EPA (Rustan et al. 1988). Studies have indicated that DHA has more inhibitory effects compared to EPA and arachidonic acid (Mikkelsen et al. 1993). In this regard, Hall et al. (2007) reported that the reduction in blood NEFA of New Hampshire laying hens fed 3% fish oil (replacing sunflower oil) can be attributed to the increased plasma EPA content. On the other hand, there are peroxisome proliferator‐activated receptors (PPARs) that are affected by different FAs and peroxisome proliferator factors, such as blood lipid‐lowering drugs (Eubank et al. 2001). There are two dominant PPAR isoforms: PPARγ, which plays a role in adipose tissue differentiation and fat storage (Hua et al. 1995), and PPARα, which increases hepatic fat oxidation through up‐regulation of carnitine palmitoyl transferase and CoA oxidase (Torra et al. 1999). Studies investigating different PUFA sources have shown that fish oil or fish oil + conjugated linoleic acid (CLA) supplementation in broiler chicken up‐regulated hepatic PPARα gene expression compared to the birds fed with CLA, soy oil and palm oil (Royan et al. 2011). Similar reports have been made in studies with rabbits (Nakatani et al. 2005), turkeys (Ding et al. 2003), rats (Issemann and Green 1990) and humans (Schmidt et al. 1992), confirming the high capacity of feeding high omega‐3 diets on activation of β‐oxidation pathways in hepatocytes. Therefore, low hepatic fat storage in fish oil‐fed birds was attributed to the activation of β‐oxidation pathways in hepatocytes, which, in turn, broke down TG into glycerol and FAs, meanwhile increasing NEFA uptake via hepatic cells (Frenkel et al. 1988, 1994). The present findings are in agreement with those of previous studies. Therefore, in‐line with Royan et al. (2011), current authors believe that increased dietary PUFAs, especially DHA and EPA, by increasing FOCS supplementation, led to the up‐regulation of hepatic PPARα. This, in turn, increased the uptake of NEFA by liver cells from the circulatory system in the FOCS_1.5_ and FOCS_2.25_ groups. However, the current study did not determine the gene expression of PPARs in liver tissue, so further research is needed in this area.

In contrast to this study, scientists reported increased NEFA in serum contents of the Tsaiya laying ducks fed 4% fish oil and/or α‐tocopherol at peak production compared to the CON (tallow), whereas the total cholesterol, phospholipids and total lipids in plasma and the yolk cholesterol and yolk weight were not different among experimental groups (Chen and Hsu 2004). The authors suggested that the decrease in plasma FAs, especially TG, was due to the inhibitory effects of fish oil *n*‐3 PUFAs on hepatic FA synthesizing enzymes. They also mentioned that peroxidation of fish oil *n*‐3 PUFAs is responsible for the inhibition of FA synthesizing enzymes (Mikkelsen et al. 1993) and that the consumption of antioxidants improves fat synthesis in the liver. They also stated that increasing α‐tocopherol in fish oil‐supplemented diets reduces NEFA in the blood, which, in turn, decreases hepatic fat synthesis. In other words, the oxidation of fish oil FAs and their inhibitory effects on fat‐synthesizing enzymes are responsible for the increased blood NEFA. To some extent, the difference in results may be attributed to the difference in experimental animals, specifically the difference in duck and laying hens’ feed composition. However, further investigations are required to understand the decrement in serum NEFA of laying hens fed FOCS or fish oil‐supplemented diets by studying the gene expression and activity of enzymes that contributed to FA synthesis. Additionally, it seems that blood sampling at the Week 4 of the experiment will help to find the most probable reason for increased production performance.

On the basis of the current findings, the authors believe that low NEFA, non‐significant difference at % EP and increased yolk percentage of FOCS_1.5_ and FOCS_2.25_ groups at Week 8 of the experiment are probably the indicators of hepatic fat deposition pathway activation (PPARγ) and transportation of produced fats, including TG, cholesterol and phospholipids, to the egg yolk via VLDLs (Finn 2007; Schneider 2009; Lin et al. 2019). Simply, feeding high *n*‐3 PUFA from the source of fish oil increased NEFA uptake from blood by hepatic cells via activation of PPARγ pathway which lead to high fat transportation to ovary in the form of VLDL.

Blood biochemical parameters evaluations in the present work demonstrated that TAC was not affected by dietary treatments. However, the highest and lowest levels of MDA were observed in the FOCS_0.75_ and FOCS_2.25_ groups, respectively. This finding contrasts with previous research, where MDA, AST and UA levels increased in 28‐week‐old laying hens (Dong et al. 2018). These authors attributed the decrease in FI (and therefore, its impact on EP and feed efficacy) to the high dietary fish oil supplementation (at the level of 8%). They also suggested that the stimulation of lipid peroxidation and impaired liver function is responsible for the increased blood MDA and hepatic enzymes, particularly AST, which in turn disrupted hepatic protein metabolism. In the most recent studies, increasing dietary PUFAs, specifically from fish oil, has been reported to increase egg yolk MDA due to their high susceptibility to oxidation. Therefore, it increased the thiobarbituric acid/MDA contents of both fresh and stored (at 4°C for 28 days) egg yolk (Kralik et al. 2020, 2021). As the most common lipid peroxidation byproduct, MDA can be measured to determine the cell membrane damages (Esterbauer and Cheeseman 1990). According to the researchers, high dietary PUFAs, especially EPA, DHA and even LA, may increase the rate of oxidation in body cells (Avula and Fernandes 1999). In this context, the experiments have shown reduced thiobarbituric acid content of egg yolk in laying hens fed fish oil or linseed oil (Scheideler et al. 1997). Hall et al. (2007) highlighted that high NEFA content in the bloodstream can stimulate oxidative stress and activate inflammatory responses, such as the transcription factor nuclear factor–kappa B in blood mononuclear cells. They suggested that increasing dietary *n*‐3 PUFA in poultry could reduce oxidative stress and inflammatory response by increasing the Leukotriene B5 eicosanoids (LBT5). These eicosanoids, derived from EPA and DHA, give rise to anti‐inflammatory prostaglandins that promote overall body health (Kinsella et al. 1990; Korver and Klasing 1997).

The authors suggest that the high EP and EW in FOCS_0.75_‐fed birds may be due to increased plasma NEFA caused by feeding high EPA and DHA‐containing diets. This likely led to increased blood MDA due to higher blood and liver PUFA contents. Interestingly, blood MDA and NEFA levels decreased with increasing FOCS supplementation levels. The current results indicate an inverse relationship between UA and MDA levels in serum, where the UA increased as the dietary FOCS level increased, except for FOCS_0.75_, where UA content was significantly reduced. As mentioned previously, these findings agree with the study of Dong et al. (2018), where the authors attributed the increased blood MDA, AST and UA to the disruption of hepatocyte structure and function, particularly protein metabolism, caused by elevated FA peroxidation. However, earlier studies with humans and animals have presented UA as an antioxidant, with its increase being a natural defence mechanism of the body against free radicals (Ames et al. 1981). Although UA has been known as the final product of protein, amino acids and purine nucleotides (guanine) degradation in poultry (Yuan et al. 1999) forming from intermediates such as inosine, xanthine and hypoxanthine during the deamination process of adenosine nucleosides (Xi et al. 2000), studies with humans have indicated that UA elevation is a natural mechanism of body against oxidative stress and aging (Nieto et al. 2000). Scientists have demonstrated that UA acts as an antioxidant in humans and any animals, especially in poultry, that are unable to synthesize ascorbic acid, which helps in radical scavenging and reducing risk of cancers (Ames et al. 1981). Further studies indicated that although the antioxidant property of UA is not as potent an antioxidant as ascorbic acid, it is successful in reducing lipid peroxidation (Frei et al. 1989). Unfortunately, there are limited studies on the effects of dietary FOCS supplementation on serum UA. On the basis of the above discussions, the authors of the present study believe that consuming high levels of *n*‐3 PUFAs, particularly EPA and DHA, through FOCS diets, especially FOCS^2.25^, increased hepatic lipid metabolism and FA uptake from the bloodstream by hepatic cells that resulted in decreased peroxidation rate and, consequently, reduced blood MDA levels. On the other hand, birds consuming high *n*‐3 PUFA diets showed increased blood UA levels, leading to consistent TAC and reduced MDA levels. Furthermore, FOCS_0.75_‐treated birds had the lowest serum UA levels compared to the other groups, which is probably thought to be responsible for their high serum MDA.

In the current experiment, the status of liver enzymes ALT, AST and ALP was also evaluated. Unfortunately, there was a high variation in the observations for ALP and AST in this study (6.370 and 7.899, respectively), making it difficult to discuss the effects of dietary treatments on liver status. The results showed that serum ALT was increased in all the FOCS‐treated groups compared to the CON. In contrast, Dong et al. (2018) reported increased serum AST by feeding laying hens with fish oil‐containing diets. Those authors did not determine the ALP and ALT levels. However, on the basis of the increased blood MDA and UA levels in the group of birds fed with 8% fish oil‐supplemented diets in their study, they attributed the high ALT levels to the increased lipid peroxidation that resulted in the functional and structural damage of hepatic cells. Furthermore, Mousavi et al. (2017) disagreed with the findings of the present study, reporting decreased serum ALP and AST levels (and no effects on ALT) in 40‐week‐old laying hens fed with 3% fish oil‐supplemented diets and CON. They suggested that the increased intake of EPA and DHA through fish oil supplementation is responsible for the reduction in liver tissue damage and lower hepatic enzymes leading to improved liver health indices (Al‐Daraji et al. 2010). AST and ALT are the primary transaminases in hepatic cells, released from cytoplasm following damage or destruction (Sun et al. 2015). Hepatic ALP has been reported to increase in 23‐week‐old laying hens fed high energy, low protein diets to induce fatty liver syndrome using 4.22% dietary soy oil (Rozenboim et al. 2016; Gao et al. 2019). Generally, on the basis of the studies mentioned above, feeding laying hens with high levels of fat sources containing high PUFA contents has been shown to increase hepatic enzyme levels in the bloodstream, which the scientists attributed it to the oxidative damage of hepatic cells. As the FA profile determinations indicated that the *n*‐3 PUFA content in the egg yolk of the FOCS_2.25_ group increased remarkably compared to the other experimental groups, increased hepatic AST levels in the serum of laying hens fed FOCS_2.25_ diets may, therefore, be attributed to the consumption of high *n*‐3 PUFA and their oxidation. However, the decreased blood MDA content of the mentioned group challenges these interpretations. For this reason, the determination of gene expression and activity of antioxidant enzymes in the liver has been suggested in the current study. In agreement with Mousavi et al. (2017) and Al‐Daraji et al. (2010), the present authors believe that the increased consumption of EPA and DHA in FOCS_2.25_‐treated birds is likely responsible for reducing blood MDA levels and maintaining TAC levels by inducing the gene expression and activity of antioxidant enzymes in the liver. Additionally, the elevated MDA and AST levels in the serum of FOCS_0.75_‐treated birds may be attributed to the insufficient consumption of EPA and DHA, resulting in inadequate induction of antioxidant enzyme production and activity in the liver.

The results also indicated that blood glucose increases by increasing dietary FOCS, especially from 1.5% to 2.25%. Earlier, Chen and Hsu (2004) found that feeding laying ducks with 4% fish oil during peak production reduced the activity of FA synthase enzymes, including Glu‐6‐phosphate dehydrogenase (G6PD), FA synthase, citrate‐ATP degrading enzyme and AAC, through its high PUFA contents. Although the former study did not determine blood glucose level, the decrease in G6PD activity suggests that, in‐line with the present findings, blood glucose increased with a fish oil‐containing diet. G6PD is a key enzyme in the hexose monophosphate pathway that uses glucose to produce energy in the form of NADPH during urgent situations (Voet and Voet 1995). There have been limited studies on the effects of dietary fish oil supplementation on blood glucose levels. However, corresponding to the present work, research on broiler chickens has shown that feeding them with fish oil supplementation led to an increase in blood glucose levels (Maniila et al. 1999). Other scientists have also reported elevated blood glucose levels in broiler chicken due to dietary fish oil supplementation (Hosseini‐Mansoub and Bahrami 2011). These findings confirmed previous experiments that reported decreased insulin secretion when high PUFA‐containing diets were consumed (Grill and Qvigstad 2000; Storlien et al. 2000). This indicates that insulin reduces the use of fat for energy and increases fat deposition when high‐energy diets are consumed. On the other hand, insulin inhibits the activity of hormone‐sensitive lipase, which is responsible for lipid breaking down for energy production (Holness et al. 2004). According to Hosseini‐Mansoub and Bahrami (2011), adding fish oil to broiler chicken diets can increase the use of fat for energy and can also affect blood glucose levels by inhibiting insulin hormones. Additionally, Warnotte et al. (1999) indicated that elevated levels of free FAs around pancreatic β cells can have an inhibitory effect on insulin hormone secretion. They also believed that the higher the degree of unsaturation of FA, the greater their inhibitory impact on insulin hormone secretion. Therefore, the inhibitory effects of dietary fish oil and sunflower oil will be more than that of tallow oil (Stein et al. 1997). Thus, in agreement with the previous studies, this study suggests that the inhibitory effects of dietary fish oil *n*‐3 PUFAs, particularly in the FOCS_1.5_ and FOCS_2.25_ groups, may be responsible for the increase in blood glucose levels due to their impact on insulin hormone secretion. It is believed that fats slow down feed passage in the gastrointestinal tract, which may lead to continued carbohydrate digestion and absorption until the end of the small intestine. This, along with providing energy for the enterocytes of the ileum, may result in increased glucose digestion and absorption, potentially explaining the higher blood glucose levels in birds fed FOCS_1.5_ and FOCS_2.25_ diets.

According to the current results, the blood phosphorus (P) level decreased in the birds fed FOCS_1.5_ and FOCS_2.25_, whereas the calcium (Ca) was alleviated in the FOCS_2.25_‐treated group compared to the other experimental groups. Limited investigations with dietary Ca salts or fish oil supplementation have studied the blood Ca and P levels. In agreement with the present findings, Dänicke (2001) reported decreased blood Ca levels by increasing dietary fat due to reduced intestinal absorption. However, Shahryari et al. (2021) recently reported no effects of dietary soy acid oil or its CS supplementation on blood Ca and P levels of broiler chickens, attributing their observation to the low dietary fat and Ca levels. Moreover, in another experiment, no impacts on blood Ca levels were reported by feeding CS (including 70% palm oil + 25% sunflower oil + 5% soy oil) supplemented diets in broiler chicken (Gado et al. 2017). In‐line with the current work, Calik et al. (2019) found that broiler chickens fed tallow CS showed decreased levels of blood Ca, P, Fe, Mn and total tibia ash content. They attributed these outcomes to the high excretion of soaps produced in the small intestine (Atteh and Leeson 1984). Zhong et al. (2014) suggested that the digestibility of dietary minerals, particularly Ca, was less affected by saponification in the small intestine when a high PUFA‐containing fat source was included in the broiler chickens’ diet. Therefore, agreeing with Zhong et al. (2014), the present authors believe that the saponification of Ca by intestinal microorganisms possibly due to the slower feed passage rate caused by dietary fats, reduced the digestibility of Ca and P in laying hens fed a high FOCS level (FOCS_2.25_) diet. Moreover, the non‐significantly higher EP in FOCS_2.25_ fed birds during the second 4 weeks of the experiment, along with the significant and non‐significant increases in eggshell‐related traits such as shell percentage, shell thickness and shell strength at Week 8 of the experiment (the week when blood samples were obtained), leads to the assumption that the high uptake of Ca by the birds’ uterus for shell formation may be responsible for the low blood Ca and P levels.

Evaluating the effects of FOCS supplementation in laying hens’ diet, this study also indicated that the eggshell percentage increased in Weeks 4 and 8. This is consistent with a study by Sattari Najaf Abadi et al. (2021), which reported increased eggshell percentage by feeding 1.5% and 3% FOCS supplementation to aged broiler breeder hens. However, a study by Dong et al. (2018) observed no significant difference in the strength and thickness of eggshells in 28‐week‐old laying hens fed 8% fish oil, soy oil and coconut oil for 20 consecutive weeks, which disagrees with the present findings. Similarly, no difference was observed in the strength and thickness of eggshells by feeding laying hens with 1.5% fish oil and 4.32% and 8.64% linseed oil (Basmacioglu et al. 2003). Moreover, no difference in eggshell traits was reported by feeding laying hens with 1.5% fish oil with or without natural and synthetic antioxidants (Orhan and Ölmez 2011). Therefore, it seems that fish oil alone is unable to affect quality traits of egg. In a study by Sattari Najaf Abadi et al. (2021), it was mentioned that previous research has shown that feeding laying hens with diets high in *n*‐3 PUFAs can affect calcium absorption and, as a result, eggshell formation (El‐Husseiny et al. 2008). The researchers stated that due to the high solubility of fat‐soluble vitamins in oils and any fat source containing high PUFAs, using these oils in the diet of laying hens can lead to increased calcium absorption from the blood, ultimately improving the quality of eggshells. But note that the effects may vary based on the type and amount of fat used in diets (Herkeľ et al. 2017). According to research by Sattari Najaf Abadi et al. (2021), companies primarily incorporate vitamin D3 into the structure of their calcium supplements. On the basis of this insight, the authors of the present study believe that the improved digestibility of calcium in the small intestine, along with increased uptake of calcium from the blood by uterine cells, is likely responsible for the enhanced percentage of eggshells. This is particularly evident in laying hens fed CON (mineral premix) and FOCS (mineral premix and CS), which resulted in greater eggshell thickness (*p* < 0.05) and shell strength (*p* < 0.05) in the FOCS_2.25_‐treated birds in this study.

Both yolk and albumen pH levels were influenced by dietary treatments at the 4‐week mark of the current experiment. The pH values for both the yolk and albumen decreased as the level of FOCS supplementation increased. This finding contrasts with the results reported by Kralik et al. (2021) who reported an increase in yolk pH; however, they agreed with our study in observing a reduction in albumen pH when fish oil and soy oil were included in the diets of laying hens. Similar outcomes were found in research that supplemented laying hens’ diets with CLA and fish oil (Alvarez et al. 2004). The authors of those studies posited that as PUFAs, particularly CLA, get incorporated into the yolk membrane (Du et al. 1999, 2000), an increase in PUFAs within the yolk membrane enhances its fluidity and permeability. This change may lead to the relocation of iron ions between the yolk and albumen, especially after the eggs have been stored for 28 days. Egg yolk contains a high concentration of iron ions, which bind to a transporting protein called phosvitin through strong ionic bonds with phosphoserine units (Causeret et al. 1991). According to the study by Causeret et al. (1991), in addition to phosvitin, iron ions also bind to other components of the yolk, such as LDL and lipovitellin, which weakens (phosvitin‐Fe^3+^) by pH decrement and causes it to become more soluble in the yolk. The released iron ions (Fe^2^⁺) can then initiate lipid peroxidation through the following reaction: LOOH + Fe^2+^ → Fe^3+^ + OH^−^ + LO^•^. Agreeing with the previous studies, Kralik et al. (2021) stated that increased albumen pH converts mucin to yolk. As the mucin entrance into the egg yolk is associated with water, reduced yolk colour was expected during storage. It is evident that the purposes and experimental diets of previous studies differ from those of the current research. Thus, the present authors believe that the decline in yolk pH is likely due to lipid peroxidation in the FOCS‐supplemented groups, particularly in the FOCS_2.25_ group, during the peak production period. Fish oil is rich in PUFAs, which are prone to peroxidation. Consequently, the reduced yolk pH may result from this lipid peroxidation. Earlier investigations have shown that when albumen pH increases, the bonds between lysosomes and ovomucin are weakened, which in turn reduces the Haugh unit (Ahn et al. 1999).

The reduction in albumen pH at Week 4 might be attributed to the direct influence of dietary FA composition on the bird's metabolism and the interactions of albumen proteins in freshly laid eggs. Some studies have reported that increasing the ratio of unsaturated FAs in the diet can improve the stability of vitelline and albumen membranes and reduce CO_2_ loss at the time of laying, which results in lower albumen pH in fresh eggs (Świątkiewicz et al. 2020). The simultaneous increase in yolk colour at Week 4 may be related to the effects of unsaturated FAs and lipophilic compounds in the FOCS on the transfer of carotenoids and pigments to the yolk (Mariod et al. 2015). Several reports have shown that omega‐3 FAs can enhance the absorption and deposition of yolk pigments in laying hens (Toomer et al. 2019). At Week 8, the increase in albumen pH might be due to production fatigue in birds and the cumulative effects of long‐term PUFA supplementation on hepatic metabolism and albumen protein composition. In addition, the significant decrease in HU observed at this stage may be linked to reduced albumen protein quality caused by the oxidative degradation of PUFAs and increased lipid peroxidation (Alagawany et al. 2019). Interestingly, yolk colour did not increase significantly at Week 8, which is consistent with previous studies suggesting that the pigmentation effects of dietary omega‐3 sources are more prominent in the short term and tend to diminish over prolonged feeding periods (Grčević et al. 2019). Overall, the observed changes in albumen pH and HU in fresh eggs throughout the feeding period may be attributed to the dynamic effects of PUFAs on internal egg quality and the structure of albumen proteins.

In this study, evaluations of egg yolk FAs in laying hens fed with FOCS‐supplemented diets showed an increase in PUFAs, particularly the *n*‐3 FAs, including EPA, DHA and ALA. Conversely, the levels of LA, the *n*‐6:*n*‐3 ratio and the LA:ALA ratio significantly decreased compared to the CON group. These results are consistent with previous research that also reported changes in FA profiles in laying hens fed diets supplemented with fish oil (Saleh et al. 2009). According to the study by Sattari Najaf Abadi et al. (2021), which used an FOCS treatment similar to that of the current study, *n*‐3 PUFAs, specifically EPA and DHA, increased, whereas *n*‐6 PUFAs, including LA, decreased in broiler breeder hens consuming diets containing up to 3% FOCS. The reduction in the *n*‐6:*n*‐3 ratio observed in their research supports the conclusion that the egg yolk FA profile can be influenced by the source of dietary fat. However, the extent of changes in each FA may differ depending on the type of fat used (Koppenol et al. 2014; Kishore et al. 2017). The egg yolk contains 67% PUFAs and 33% SFAs (Fernandes et al. 2018). PUFAs are primarily produced by hepatic enzymes through the elongation and desaturation processes. For example, ALA is converted into EPA and DHA, whereas LA is transformed into arachidonic acid (Koppenol et al. 2014; Cherian and Quezada 2016). Due to the competition among lipid metabolism enzymes on substrates, Sattari Najaf Abadi et al. (2021) suggested that *n*‐3 FAs take precedence over *n*‐6 FAs. Therefore, feeding fat sources that are rich in *n*‐3 PUFAs can increase DHA levels while decreasing the presence of arachidonic acid in the yolk. In the production pathways for DHA and arachidonic acid, the Δ‐6 desaturase has been identified as the rate‐limiting enzyme competing with ALA and LA for its substrate. Consequently, when a high level of LA is consumed, more arachidonic acid and its derivatives are formed (Cherian 2008; Koppenol et al. 2014; Cherian and Quezada 2016). According to a recent meta‐analysis by Irawan et al. (2022), researchers found that feeding laying hens fish oil significantly increases the levels of *n*‐3 PUFA and decreases levels of *n*‐6 PUFAs in egg yolk FA contents. In‐line with this study and previous findings, the researchers demonstrated that a diet high in ALA and low in LA leads to a linear increase in DHA in yolk FA contents (Huang et al. 2018; Omri et al. 2019; Aguillón‐Páez et al. 2020; Lee et al. 2021). These observations support the idea that ALA serves as a precursor for the production of EPA and DHA. When laying hens are fed a fat source rich in ALA, the enzyme Δ‐6 desaturase facilitates the conversion of ALA to EPA by removing a hydrogen atom and adding a carbon bond during the elongation process. Subsequently, the EPA is converted to DHA through another elongation and desaturation step (Fraeye et al. 2012). However, later studies revealed that the varying LA content in different fat sources affects the efficiency of converting ALA to DHA, leading to discrepancies in findings across various studies (Ehr et al. 2017). The tendency for ALA levels to be higher than those of LA has been noted; however, Fraeye et al. (2012) suggested that this variation may be attributed to the competition between ALA and LA for the substrate site of Δ‐6 desaturase. As a result, a decreased LA:ALA ratio can disrupt DHA production. In a comparison of three fat sources—fish oil, linseed oil and microalgae—research indicated that the conversion efficiency of ALA to DHA in laying hens supplemented with fish oil was greater than in those given the other fat sources. This increased efficiency is primarily due to the higher DHA content found in fish oil (Rizzi et al. 2009). Further studies have supported these findings, showing that feeding laying hens diets supplemented with fish oil leads to increased *n*‐3 PUFAs, particularly EPA and DHA (Saleh et al. 2010; Ebeid 2011; Kralik et al. 2021). Therefore, in‐line with previous research, the authors of this study believe that elevating dietary levels of fish oil contributes to higher *n*‐3 PUFA deposition in egg yolk, largely due to the influence of the high *n*‐3 PUFA content in fish oil on Δ‐6 desaturase activity.

## 5 Conclusions

The results of this study recommend a dietary supplementation of 0.75% FOCS to enhance production performance and improve egg quality traits during the peak production period for laying hens. Additionally, for the enrichment of egg *n*‐3 PUFA, a supplementation of 2.25% FOCS is suggested to harness its health‐promoting benefits.

## Author Contributions

**Hamze Ghaderi‐Chaparabad**: investigation, data curation. **Seyyed Ali Mirghelenj**: supervision, conceptualization, project administration, formal analysis. **Sina Payvastegan**: validation, visualization. **Hamed Khalilvandi‐Behroozyar**: methodology, project administration. **Amir Mosayyeb Zadeh**: roles/writing – original draft, writing – review and editing.

## Ethics Statement

The authors confirm that the ethical policies of the journal, as noted on the journal's author guidelines page, have been adhered to and the appropriate ethical review committee approval has been received. The authors confirm that they have followed Animal Care Committee and Animal Research Ethics Board approval from the Department of Animal Science, Urmia University, Iran, under the protocol number of IR‐UU‐AEC 1344/PD/3 throughout the study.

## Conflicts of Interest

The authors declare no conflicts of interest.

## Data Availability

The data that support the findings of this study are available from the corresponding author, Seyyed Ali Mirghelenj, upon reasonable request.
